# Sensor Fusion for Target Detection Using LLM-Based Transfer Learning Approach

**DOI:** 10.3390/e27090928

**Published:** 2025-09-03

**Authors:** Yuval Ziv, Barouch Matzliach, Irad Ben-Gal

**Affiliations:** 1 Department Industrial Engineering, Tel-Aviv University, Tel-Aviv 6997801, Israel; 2 LAMBDA Laboratory, Tel-Aviv University, Tel-Aviv 6997801, Israel

**Keywords:** search and detection, sensor fusion, mobile agents, neural networks, deep learning, large language models, transfer learning, distillation

## Abstract

This paper introduces a novel sensor fusion approach for the detection of multiple static and mobile targets by autonomous mobile agents. Unlike previous studies that rely on theoretical sensor models, which are considered as independent, the proposed methodology leverages real-world sensor data, which is transformed into sensor-specific probability maps using object detection estimation for optical data and converting averaged point-cloud intensities for LIDAR based on a dedicated deep learning model before being integrated through a large language model (LLM) framework. We introduce a methodology based on LLM transfer learning (LLM-TLFT) to create a robust global probability map enabling efficient swarm management and target detection in challenging environments. The paper focuses on real data obtained from two types of sensors, light detection and ranging (LIDAR) sensors and optical sensors, and it demonstrates significant improvement in performance compared to existing methods (Independent Opinion Pool, CNN, GPT-2 with deep transfer learning) in terms of precision, recall, and computational efficiency, particularly in scenarios with high noise and sensor imperfections. The significant advantage of the proposed approach is the possibility to interpret a dependency between different sensors. In addition, a model compression using knowledge-based distillation was performed (distilled TLFT), which yielded satisfactory results for the deployment of the proposed approach to edge devices.

## 1. Introduction

The detection of hidden static or mobile targets is a fundamental problem in various fields, from military operations to search-and-rescue missions [1,2]. Classical search theory, established by Koopman [3] and developed by Stone [1] and Washburn [2], originally focused on optimal search effort distribution to maximize target detection probability. Modern applications have evolved to encompass autonomous agent swarms with sophisticated sensor systems, introducing complexities in inter-agent communication, distributed decision-making, and sensor uncertainty handling. To address these challenges, various approaches have been developed, including heuristic algorithms utilizing expected information gain (EIG), center of view (COV), and center of gravity (COG) with Bayesian inference [4] and deep Q-learning methods demonstrating superior performance in noisy environments [5,6]. These methods assume error-prone sensors, susceptible to both false positive (type I) and false negative (type II) errors.

A significant limitation of the application of these methods is the reliance on theoretical sensor models. While these models are valuable for conceptual development, their direct applicability to real-world scenarios is often hampered by the complex and unpredictable nature of actual sensor performance, especially under adverse environmental conditions. Real sensors, such as light detection and ranging (LIDAR) sensors and optical cameras, possess distinct advantages and disadvantages. Optical sensors offer high-resolution imagery and excel in object classification but miss depth information and degrade in low light. LIDAR, conversely, provides high accuracy in distance measurements and performs well under low-light conditions and adverse weather but typically cannot classify objects inherently. The integration of these complementary sensor types is crucial for robust detection in diverse environments.

The widely known method of sensor fusion is based on convolutional neural networks (CNNs) [7], including ResNet architecture [8]. These models raised new possibilities for learning sensor data and providing impressive performance. However, CNNs might present challenging issues such as vanishing gradients or exploding gradients. ResNet addresses these difficulties by incorporating “shortcut connections” that enable residual learning, thereby facilitating the optimization of deep networks [9].

This study proposes a novel sensor fusion approach that utilizes real sensor data (LIDAR and optical cameras) within an LLM-based transfer learning (LLM-TLFT) framework. This allows for the creation of a comprehensive and accurate global probability map enabling effective collective decision-making in the swarm of autonomous agents. This study aims to develop algorithms for generating a global centralized probability map to enable effective swarm management of autonomous agents by fusing data from different types of real sensors.

Recently, the application of large language models (LLMs) has emerged as a promising avenue for complex data processing tasks beyond natural language. For sensor fusion applications specifically, deep transfer learning with versions such as GPT-2 or CLIP models, which are originally designed for natural language processing or finding similarities between text and images, have been adapted for industrial sensor fusion tasks [10,11]. This study showcased how the powerful feature learning capabilities of LLMs can be adapted to process diverse sensor data, reducing the need for extensive training data and enabling automatic feature extraction. Building on foundational work, pioneering research by Radford et al. demonstrated how contrastive pre-training can learn transferable visual representations from natural language supervision, establishing fundamental principles for cross-modal learning [12]. This breakthrough showed that models trained on text–image pairs can develop robust visual understanding capabilities that transfer effectively to downstream tasks. Subsequent research has further explored LLM applications in various domains, including reinforcement learning optimization where human feedback alignment has been successfully applied to improve code generation performance [13].

## 2. Sensors and Generating Probability Maps

Effective target detection in dynamic and uncertain environments depends on the quality of information gathered by the agents’ sensors and on the quality of information processing and understanding of the search domain. The suggested system leverages the complementary strengths of optical cameras and LIDAR sensors to achieve this.

### 2.1. Sensor Specifications

Autonomous agents are equipped with a suite of sensors designed to perceive their environment. Optical sensors yield rich visual data, allowing for high-resolution imagery and effective object classification due to their high angular accuracy. However, they inherently cannot provide information about the depth or range of the objects in the image, and the performance of such sensors significantly degrades under low-light conditions or adverse weather. Furthermore, processing high-resolution image data demands substantial computational resources.

Conversely, LIDAR sensors provide accurately measured distances and precise depth and range information. Their effectiveness is maintained even under low-light conditions and adverse weather, making them a robust choice for environmental sensing. Nevertheless, LIDAR systems can be more expensive than other sensor technologies, and the raw point-cloud data they generate typically lacks semantic classification, thus requiring additional processing for semantic understanding.

### 2.2. Search Area and Probability Maps

Following the notation presented in [4], assume that the search domain is represented as a finite set of cells, $C=\{c_{1},c_{2},\ldots ,c_{n}\}$, forming a gridded two-dimensional area. Each cell $c_{i}\in C$ can either be empty $\left(s(c_{i})=0\right)$ or occupied $\left(s(c_{i})=1\right)$ by a target.

The state $s(c_{i})$ of the cell is a discrete random variable, and for any cell $c_{i}$, the probabilities of its states are complementary, that is, $Pr\{s(c_{i})=0\}+Pr\{s(c_{i})=1\}=1$.

The agents’ collective knowledge about target locations is represented by a shared “probability map”, where $p_{i}=\mathrm{Pr}\left\{s\left(ci\right)=1\right\}$ is the probability that cell $c_{i}$ is occupied.

Assume that the agent is in the cell $c_{j}$. Then the probability that the agent receives a signal $\tilde{s}$ from a cell $c_{i}$ is

(1) $$p\left(\tilde{s}\left(c_{i}\right)\right)=\frac{\dot{\mathrm{Pr}\left\{s\left(c_{i}\right)=1|\tilde{s}\left(c_{i}\right)=1\right\}}\mathrm{Pr}\left\{\tilde{s}\left(c_{i}\right)=1|s\left(c_{i}\right)=1\right\}}{\sum_{c_{i}}\dot{\mathrm{Pr}\left\{s\left(c_{i}\right)\right\}}\mathrm{Pr}\left\{\tilde{s}\left(c_{i}\right)=1\right|s(c_{i})\}}$$

The signals received by the agents are called alarms, which with respect to their truth are defined as true and false alarms.

Receiving information and updating the shared probability map are crucial for cooperative search. Each agent senses the environment by receiving true and false alarms and updates its understanding of target locations by contributing to the probability map shared among the agents in the group.

### 2.3. Sensor Fusion and Probability Map Hierarchy

The proposed employs a two-tier hierarchical structure for probability map organization [4]:

- Sensor map: Each kth sensor generates a sensor probability map $P^{sensor}(k)$ representing the likelihood of targets in each cell based on its own readings of the kth sensor.
- Agent map: Information from the sensors on the jth agent is fused to create an agent-level probability map $P^{agent}(j)$. This fusion process is crucial for leveraging the diverse capabilities of different sensor types.
- Global map: This probability map is obtained by merging the agent maps and represents common knowledge of the group of agents. In the considered case of the search by a single agent, a global map is equivalent to the agent map and is called a global agent map.

**Figure 1:**
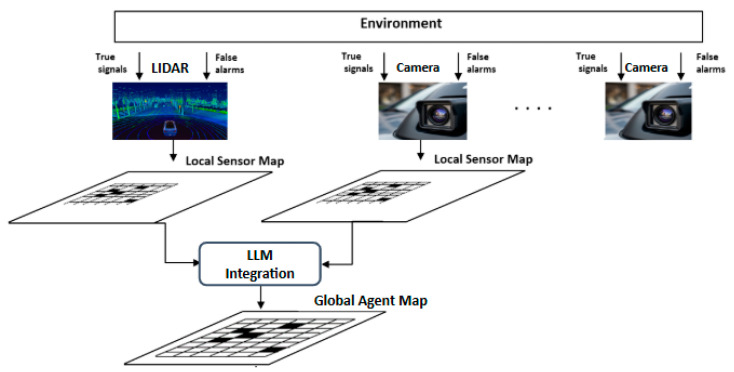
Hierarchy of the probability maps and sensor fusion process.

The hierarchy of the probability maps created based on the sensor data is shown in Figure 1.

Local sensor maps are independently generated from LIDAR and camera data, capturing both true signals and false alarms from the environment. These individual maps are then intelligently fused by the LLM integration module to create a single, robust global agent map that represents the agent’s unified perception.

The fusion of sensor maps into agent-level (global) maps is performed according to the following procedure: denote by $p_{s_{i}}^{sensor}\;(j,k)$ a probability that the cell $c_{i}$ is occupied as it is estimated by the kth sensor installed on the jth agent.

Let ${\tilde{s}}_{jk}$ be a signal received from the kth sensor of the jth agent. Fusing information from the sensors of the agent results in the agent’s probability map, in which the probability of target’s location in the cell $c_{i}$ is

(2) $$p_{s_{i}}^{sensor}\;(j,k)=Pr\{s_{i}=1\;|\;{\tilde{s}}_{jk}(c_{i})\}=\frac{\left(\mathrm{Pr}\left\{s_{i}=1\right\}\;\cdot \;\mathrm{Pr}\left\{{\tilde{s}}_{jk}\left(c_{i}\right)=1\;\right|s_{i}\}\right)}{\sum_{s_{i}}\left(s_{i}Pr\{s_{i}\}\;\cdot Pr\{{\tilde{s}}_{jk}(c_{i})=1\;|\;s_{i}\}\right)}.$$

Assume that the sensors’ readings are independent and for each sensor, the probability of the cell’s state is defined. Since the sensors are independent, the joint probability of the cell’s state provided by m sensors is defined as follows:

(3) $$P_{s\left(c_{i}\right)=1}^{agent}\left(j\right)=\frac{\prod_{k-1}^{m}p_{s_{i}}^{sensor}\;(j,k)}{\prod_{k-1}^{m}p_{s_{i}}^{sensor}\;(j,k)+\;\prod_{k-1}^{m}\left(1-p_{s_{i}}^{sensor}\;(j,k)\right)}.$$

This hierarchical fusion allows for modular and computationally efficient aggregation of the data enabling decision-making at various levels of abstraction.

Note that while the global map provides a comprehensive view, its real-world implementation might involve communication and computational challenges, as it typically requires a central unit to collect and process data from all agents.

## 3. Methodology

The proposed method utilizes large language models (LLMs) and transfer learning (TL) to enhance the creation of a global probability map in the collective search by a group of several agents. This approach directly addresses the limitations of theoretical sensor models through the utilization of real sensor data and exploitation of the semantic understanding and capabilities of contextual reasoning by LLMs.

In this method, it is assumed that initial probability maps exist and are well-defined, and it is required that the method effectively detects both static and mobile targets, that the number of targets can change during the search, and that the search is conducted in highly noisy environment with the detection errors of the first and the second types.

### 3.1. Sensor Data Preprocessing and Probability Map Transformation

Prior to fusion, raw data acquired from LIDAR and optical sensors are subjected to several specific preprocessing procedures.

For optical sensors, we utilize an object detection model, namely the DETR model [14], to identify potential targets. Subsequently, given the average dimensions and standard deviation of each object type in our dataset, the pinhole camera model distance estimation technique is applied to localize these detected objects in 3D space [15]. The output is transformed into an optical sensor-specific probability map, representing the likelihood of objects within grid cells based on visual cues and estimated distances.

For LIDAR sensors, the intensities of LIDAR point cloud are averaged for each grid cell. These average intensities are converted into probabilities, where the higher intensities indicate the higher likelihood of the presence of an object.

**Figure 2:**
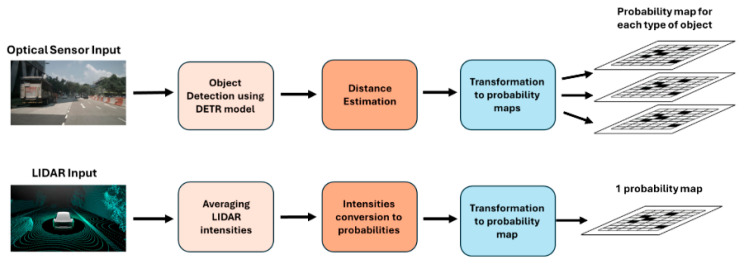
The scheme of data preprocessing for optical and LIDAR sensors. The optical stream uses a DETR model for object detection and subsequent distance estimation to create separate probability maps for each object type. Simultaneously, the LIDAR stream converts averaged point cloud intensities into probabilities to generate a single, unified probability map.

The scheme of these processes is shown in Figure 2.

### 3.2. LLM-Based Transfer Learning (LLM-TLFT)

The core innovation of the proposed method lies in the application of the LLMs for sensor fusion via transfer learning (TL). Transfer learning is a powerful paradigm that leverages knowledge gained from a pre-trained model on a specific task to improve learning in a new related domain. This approach proves particularly beneficial for the present application, given the constraints imposed by limited spatial training data, the novelty of cross-domain applications (fusing disparate sensor modalities), and the pursuit of computational efficiency.

LLMs, leveraging their inherent semantic understanding and contextual reasoning abilities, are well-suited to interpret and integrate the diverse information streams from different sensors. By fine-tuning the pre-trained LLM, we aim to enable it to effectively fuse the probability maps generated from optical and LIDAR data, resulting in a superior global probability map.

The proposed LLM-TLFT process includes the following stages:

- Data preprocessing: As described above, raw sensor data are converted into probability maps.
- Embedding layer: These probability maps are then fed into an embedding layer, which transforms the high-dimensional input into a format suitable for the LLM.
- Flatten layer: The output from the fine-tuned LLM is passed through a flatten layer.
- Output (global map): The final output is the refined global probability map, which consolidates all sensor information.

**Figure 3:**
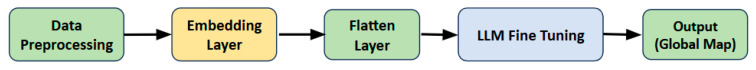
An illustration of the LLM-TLFT approach. The model takes the preprocessed probability maps and passes them through an embedding and a flatten layer to create a suitable input format. This data is then used to fine-tune the large language model, which processes the information to output the final, refined global map.

These stages are summarized in Figure 3.

In the suggested methods, the chosen LLM is Llama 3.2 1B (lightweight multimodal large language model) developed by Meta with 1 billion parameters [16]. This model is particularly attractive due to its efficiency and ability to handle both text and vision-based AI applications, making it versatile for visual Q&A and document understanding. Furthermore, its architecture incorporates optimizations like grouped-query attention (GQA), which significantly improves inference speed and reduces the memory footprint compared to traditional models, a critical factor for near-real-time sensor fusion tasks.

During the fine-tuning stage, we adopted a targeted training strategy by unfreezing only the last five layers of the pre-trained Llama 3.2 model, which empirically yielded the best performance. This technique allows the model to tailor its most abstract, high-level feature representations to the specific nuances of the sensor fusion task while retaining the robust, general knowledge from its foundational layers. To handle the significant class imbalance inherent in our dataset—where non-target cells vastly outnumber target cells—we implemented a weighted binary cross-entropy (BCE) loss function:

(4) $$BCE\;Loss=-\frac{1}{N}\sum_{i=1}^{N}\left[y_{i}\log\left(p_{i}\right)+\left(1-y_{i}\right)\log\left(1-p_{i}\right)\right]$$

Assuming that *N* is the number of observations, $y_{i}$ is the actual binary label (0 or 1) of the ith observation and $p_{i}$ is the predicted probability of the ith observation being in class 1.

This approach assigns a greater penalty to false negative errors by 50 times than to false positive errors, thereby prioritizing the detection of true targets, which is critical for enhancing recall in search and detection applications.The key advantage of LLMs in sensor fusion lies in their ability to capture complex dependencies between probability distributions from different sensors through their attention mechanisms. Unlike traditional fusion methods that assume sensor independence (as in Equation (3)), LLMs model the statistical relationships between LIDAR and optical probability maps through several mechanisms.

#### 3.2.1. Attention Mechanism

The multi-head attention mechanism in Llama 3.2 computes cross-modal attention weights between LIDAR and optical sensor probability maps [17]:

(5) $$Attention\left(Q,K,V\right)=softmax(\frac{QK^{T}}{\sqrt{d_{k}}})V\;$$

where *Q*, *K*, and *V* are query, key, and value matrices derived from the embedded probability maps. Crucially, our approach uses cross-modal attention, where LIDAR embeddings serve as queries (*Q*), while optical embeddings provide both keys (*K*) and values (*V*), enabling the model to learn sensor dependencies rather than treating modalities independently. Here are some examples for pattern learning:

- High probability values in LIDAR maps correlating with high probability in optical maps indicates reliable detection.
- Discordant probabilities (high optical, low LIDAR, or vice versa) trigger learned resolution strategies.
- Spatial probability gradients across both maps reveal object boundaries and extent.

#### 3.2.2. Learning Sensor-Specific Reliability Patterns

Through fine-tuning, the LLM learns implicit sensor characteristics, distinguishing between optical sensors’ sharp probability peaks and LIDAR’s smoother distributions. The model identifies inter-sensor disagreement patterns and develops conditional reliability assessment, using one sensor’s patterns to appropriately weight the complementary sensor during fusion.

### 3.3. Knowledge-Based Distillation

To make the advanced LLM-based transfer learning (LLM-TLFT) approach suitable for deployment on resource-constrained edge devices, we used knowledge-based distillation [18] as a model compression technique. This technique is a crucial model compression strategy that transfers the “knowledge” from a larger, more complex “teacher” model (in this case, the full LLM-TLFT model) to a smaller, more efficient “student” model.

The training process employed a composite loss function that combined two distinct objectives, balanced by a hyperparameter α set to 0.4. The primary component was a distillation loss, which used the mean squared error (MSE) to minimize the difference between the student’s output probabilities and the “soft” probability distributions generated by the teacher model. This encourages the student to learn the nuanced, inter-class relationships captured by the larger model. The second component was student loss, which used the same weighted binary cross-entropy (BCE) function from the teacher’s training, comparing the student’s predictions directly against the ground-truth labels. The final training objective was a weighted sum$:\;L_{total}=\alpha \cdot L_{BCE\;Loss}+(1-\alpha )\cdot L_{MSE}$. This formulation places a slightly greater emphasis on matching the teacher’s outputs while still grounding the student’s learning in the true data.

The objective is to significantly achieve a substantial reduction in the computational memory requirements of the LLM while preserving a substantial portion of its performance. This allows for real-time inference and operation on hardware with limited processing power, making the sophisticated sensor fusion capabilities of LLM-TLFT practical for autonomous agents and other end devices.

The successful application of distilled TLFT, as evidenced by its satisfactory results, underscores its importance in bridging the gap between high-performance, large-scale models and their real-world applicability in scenarios demanding efficiency.

The selection of vision transformer (ViT) [19] as the student model architecture is motivated by theoretical considerations of knowledge transferability and architectural alignment. The fundamental architectural homology between ViT and the Llama 3.2 teacher model—specifically, their shared reliance on multi-head self-attention mechanisms—facilitates more effective knowledge distillation. This structural correspondence is particularly significant in the context of sensor fusion, where the teacher model encodes complex inter-sensor dependencies through learned attention weights. The attention-based formulation enables the student model to preserve the teacher’s capacity for modeling long-range spatial correlations between disparate sensor modalities, a capability that would be inherently limited in architectures employing local receptive fields.

Moreover, ViT presents satisfactory trade-off between model expressiveness and computational efficiency for edge deployment scenarios. Through architectural modifications including reduced transformer depth (from 32 to 6 layers), decreased embedding dimensionality, and fewer attention heads, the student model achieves a compression ratio of approximately 200:1 (from 1B to 5M parameters) while maintaining the fundamental computational paradigm of the teacher. This preservation of the attention-based processing pipeline, despite significant parameter reduction, ensures that the distilled model retains the capacity to represent the complex probabilistic relationships learned by the teacher model, thereby enabling effective sensor fusion on resource-constrained devices.

### 3.4. Comparison with Other Sensor Fusion Methods

To contextualize the performance of the suggested LLM-TLFT approach, we evaluate it against other established sensor fusion methods:

- Independent opinion pool- (Bayesian approach), as discussed in Section 2.3, combines probabilities from independent sensors and agents [4]. It sounds mathematical under assumptions of independence but may not fully capture complex relationships or non-linear interactions between sensor modalities.
- Convolutional neural networks- (CNNs) are well-known for their ability to learn important features automatically and capture spatial relationships within sensor data [20]. We employ ResNet architecture for its proven effectiveness in various computer vision tasks [7]. While CNNs are powerful, they might not inherently possess the semantic understanding of LLMs, which can be beneficial for fusing diverse sensor data streams that represent different types of information (e.g., depth from LIDAR, object class from camera).
- Transfer learning using GPT-2 [10] is an old-versioned pretrained model for sensors fusion using the transfer learning technique. This model, in contrast to the proposed model, was trained only on textual data but not on visual data like Llama-3.2.

To quantitatively assess the performance of the proposed methodology and benchmark it against alternative approaches, we utilize the following evaluation metrics:

- Loss measures the discrepancy between the predicted and actual global probability maps during training.
- Precision, recall, and F1 score are standard metrics for classification tasks, assessing the accuracy of identified targets.
- Area under the curve (AUC) evaluates the overall performance of the model across various classification thresholds.

## 4. Implementation Example and Experimental Setup

The proposed method was implemented and evaluated on the Nuscenes dataset, a real-world dataset for autonomous driving research [21].

The dataset utilized is a large-scale collection specifically designed for autonomous driving research, gathered from diverse locations including Boston and Singapore. It comprises 1.4 million camera images from six cameras and corresponding LIDAR data. The dataset includes annotations for 23 object classes with 3D bounding boxes, providing ground truth for performance evaluation.

In the experiments, we focused on three object types, namely trucks, cars, and pedestrians. The experiments were conducted on a computational platform equipped with NVIDIA GPU P100 and an Adam optimizer. For the LLM-TLFT approach, we specifically used Llama 3.2—1B parameters. The training details are summarized in Table 1.

## 5. Results

Experimental results obtained using the proposed methodologies LLM-TLFT and the approaches indicated above, evaluated using the above metrics, are summarized in Table 2.

The obtained results indicate the superior performance of the LLM-TLFT approach, particularly the classic TLFT, for all evaluation metrics.

- Recall: TLFT achieves a recall of 0.942, significantly outperforming other methods, suggesting its ability to identify almost all true targets. This capability is crucial in search and detection tasks, where failing to identify targets can have significant negative implications. Distilled TLFT achieves outperforming results as well.
- Precision: While TLFT demonstrates very high precision, distilled TLFT achieves a precision of 0.644, indicating a decrease in the ability to distinguish between false alarms and true signals. However, the precision in both LLM-TLFT and distilled TLFT are significantly higher compared to other methods, reflecting their inability to handle the noise received by LIDAR.
- F1 Score and AUC: Both TLFT and distilled TLFT show remarkably high F1 scores (0.959 and 0.755, respectively) and AUC values (0.971 and 0.956, respectively), further confirming their overall effectiveness and robust performance.
- Loss: Significantly lower loss values for TLFT (0.09) and distilled TLFT (0.08) compared to other methods underscore their superior learning capabilities and ability to accurately model complex relationships in sensor data.

**Figure 4:**
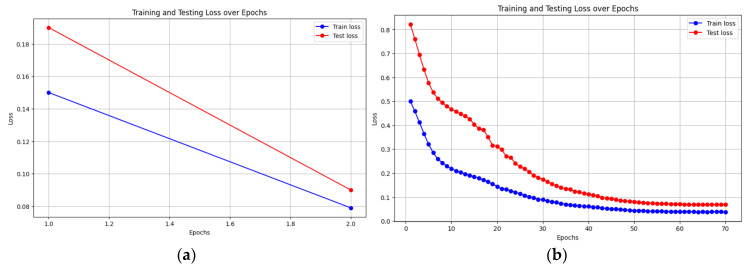
Training and testing loss curves versus epochs for the full and distilled models. Graph (**a**) displays the loss for the main TLFT model, while graph (**b**) shows the loss for the compressed distilled TLFT model. The exponential decrease and convergence of the loss curves in both plots indicate effective learning and successful generalization on the test dataset.

Figure 4 illustrates the training and testing loss over epochs for the LLM-TLFT model. Exponential decrease and convergence of the loss indicate effective learning and generalization.

The key performance metrics per class in the TLFT model and the distilled TLFT model are presented in Table 3.

The TLFT model demonstrates a recall-optimized classification strategy with consistently high sensitivity (0.917–0.945) across all classes, though the Car class shows notable precision challenges (0.855), suggesting systematic misclassification of other objects as cars.

Despite these precision–recall trade-offs, the tight F1 score clustering (0.898–0.916) indicates balanced overall performance, with Pedestrian achieving optimal discrimination and Car requiring potential feature refinement to reduce false positive rates.

The distilled TLFT model has satisfactory performance in terms of recall and precision, considering its number of parameters.

However, the car class has the worst precision and the highest precision–recall difference, which indicates unstable classification ability.

Overall, the recall score decrease compared to the TLFT model is negligible, but the precision score decrease is significant.

### 5.1. Root Cause Analysis of Distillation Performance Loss

The significant precision degradation observed in the distilled TLFT model (from 0.864 to 0.644) stems from several interconnected factors inherent to the knowledge distillation process. The compression from 1 billion to 5 million parameters creates knowledge bottlenecks, where the student model cannot fully capture the teacher’s sophisticated sensor fusion capabilities, particularly the nuanced relationships between LIDAR intensity patterns and optical object classifications.

### 5.2. Class-Specific Performance Analysis:

- Car Class: The “Car” class exhibits the most dramatic precision degradation (0.855 to 0.602). This substantial loss can be attributed to car variable LIDAR reflectance characteristics and their dual nature as both dynamic and static objects, creating complex sensor signatures that the compressed model struggles to interpret accurately.
- Pedestrian Class: The “Pedestrian” class shows significant recall degradation (0.934 to 0.762), indicating that the compressed model has difficulty detecting smaller targets that may occupy partial grid cells or exhibit weaker sensor signatures. The reduced model capacity particularly impacts the detection of these subtle targets.
- Truck Class: While not explicitly detailed in the original analysis, the Truck class maintains relatively stable performance, likely due to their larger size and more consistent LIDAR signatures compared to the other object classes.

## 6. Discussion

This paper introduces an effective methodology for multi-sensor target detection, addressing the critical need for robust sensor fusion using real-world sensor data.

By integrating LLM-based transfer learning, the proposed methodology demonstrates the capacity to generate more accurate and reliable global probability maps, which are essential for efficient swarm management of autonomous agents.

Empirical results show that our LLM-TLFT approach significantly outperforms traditional methods like the Independent Opinion Pool and CNNs in terms of key performance metrics, highlighting its potential for real-world applications in complex and noisy environments.

The use of complementary sensor types, such as LIDAR and optical cameras, coupled with the semantic understanding capabilities of LLMs, leverages the advantages of each sensor for superior probability map fusion. Furthermore, the satisfactory performance of distilled TLFT makes our solution particularly suitable for real-time operational systems with computational constraints.

The creation of these precise probability maps opens the door for a wide variety of applications, including but not limited to military operations, smart city infrastructure management, and advanced autonomous vehicle navigation.

The suggested approach advances the field by moving beyond theoretical sensor models and providing a practically viable solution for complex detection problems, and it forms a basis for future advancements in sensor fusion and autonomous agent coordination in several areas. For example, it can be extended for sensor fusion in multi-agent LLMs, where multiple LLMs communicate and collaborate for collective intelligence, combining instruction LLMs for explainability, which can enhance the transparency and interpretability of fusion decisions.

## 7. Conclusions

This paper examines sensor fusion methodologies for optical and LIDAR sensors and suggests the LLM-based transfer learning method for multi-sensor data fusion in target detection.

Experimental results demonstrate the superior performance of the proposed method, especially in comparison with the known Independent Opinion Pool and traditional convolutional neural networks (CNNs), even with the most efficient ResNet architecture.

The LLM-TLFT model consistently achieved higher recall, precision, F1 score, and AUC values, along with significantly lower loss, highlighting its ability to effectively integrate real-world LIDAR and optical sensor data and accurately model complex physical relationships of the sensor input.

Notably, the LLM-TLFT’s high recall (0.942) is crucial for search and detection tasks where missing targets might have severe consequences, while its strong precision indicates a superior ability to distinguish between true signals and noise compared to other methods.

Even the compressed version of the suggested method, the distilled TLFT, maintained impressive performance, showcasing the effectiveness of knowledge distillation for deployment on resource-constrained devices.

The ability of LLM-TLFT to leverage the semantic understanding and contextual reasoning capabilities of large language models is a distinct advantage over CNNs.

## Figures and Tables

**Table 1:** Model size and running time at the training stage in the experiments.

Method	# of Model Parameters	Running Time
Transfer Learning Fine-Tuning (TLFT)	1B	7 min
Zhang (GPT-2)	147M	5 min
CNN	1.9M	3 min
Distilled TLFT	5M	1 min
Independent Opinion Pool	4800	<1 min

Note: Hyperparameters were optimized using Optuna [22] with 50 trials and optimization algorithm named tree-structured Parzen estimator (TPE) (default). Note that this configuration was conducted for all the methods except for Independent Opinion Pool (since this is a deterministic method without learning). Batch size = [8,16,32], LR = [1 × 10^−6^, 5 × 10^−6^, 1 × 10^−5^, 5 × 10^−5^, 1 × 10^−4^, 5 × 10^−4^, 1 × 10^−3^, 5 × 10^−3^, 1 × 10^−2^, 5 × 10^−2^, 1 × 10^−1^, 5 × 10^−1^]; number of epochs = [1…30]. This table contrasts the computational cost for each method, highlighting the difference between the resource-intensive TLFT model (1B parameters, 7 min) and the highly efficient, compressed distilled TLFT (5M parameters, 1 min).

**Table 2:** Performance comparison of sensor fusion methods. The results indicate superior performance of the proposed methods. The full TLFT model achieves the highest scores in all categories, including a recall of 0.942 and AUC of 0.971. The compressed distilled TLFT also maintains robust performance, significantly outperforming traditional methods like the CNN and Independent Opinion Pool.

Method	Recall	Precision	F1-Score	AUC	Loss
Independent Opinion Pool	0.565	<0.191	0.285	0.719	4.889
CNN	0.729	<0.156	0.257	0.812	0.588
Zhang (GPT-2)	0.462	<0.224	0.292	0.713	0.639
TLFT	0.942	0.864	0.901	0.971	0.08
Distilled TLFT	0.915	0.644	0.755	0.956	0.09

**Table 3:** Performance metrics for the TLFT model and the distilled TLFT model. The table details the performance trade-offs resulting from model compression. While the full TLFT model demonstrates consistently high F1 scores across all object classes, the distilled TLFT model shows a significant drop in precision for the “Car” class (0.602) and recall for the “Pedestrian” class (0.762), highlighting the specific impacts of the distillation process.

	TLFT				Distilled TLFT	
Class	Precision	Recall	F1-Score	Precision	Recall	F1-Score
Truck	0.906	0.917	0.911	0.817	0.905	0.859
Car	0.855	0.945	0.898	0.602	0.967	0.742
Pedestrian	0.899	0.934	0.916	0.890	0.762	0.821

## Data Availability

The data presented in this study are available in Nuscenes at https://nuscenes.org/nuscenes, reference number [19]. These data were derived from the following resources available in the public.
